# INNOVATIVE RECANALIZATION TECHNIQUE USING DIATHERMIC PUNCTURE UNDER COLANGIOSCOPY FOR COMPLETE BILIARY STRICTURE

**DOI:** 10.1590/S0004-2803.24612025-135

**Published:** 2026-10-26

**Authors:** Martin GUIDI, Julio DE MARIA, Virginia Machado, Gisele Salome LOPEZ

**Affiliations:** * Hospital El Cruce, Florencio Varela, Buenos Aires Province, Argentina

## Case presentation

Postoperative biliary strictures are the most common cause of benign biliary strictures. Endoscopic retrograde cholangiopancreatography (ERCP) with stent placement is considered the first-line treatment. Few endoscopic techniques have been reported for complete strictures, as the guidewire often fails to pass through.

A 68-year-old man who had previously undergone a converted subtotal cholecystectomy for Mirizzi syndrome type III was referred for ERCP after outpatient laboratory tests indicated signs of cholestasis. Magnetic resonance cholangiopancreatography revealed a complete short stricture of the common bile duct (Figure 1). During ERCP, a complete stricture was identified that prevented the passage of the guidewire, despite the use of digital single-operator cholangioscopy (Figure 2). A decision was made to perform endoscopic biliary recanalization under direct retrograde cholangioscopic and radiological guidance.

**FIGURE 1:**
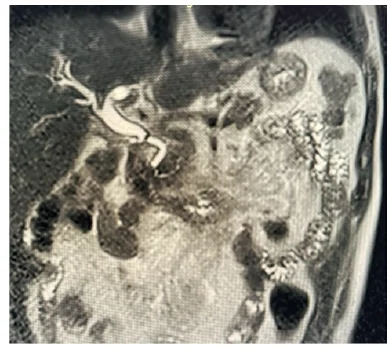
CRNM showing complete biliary stricture

**FIGURE 2:**
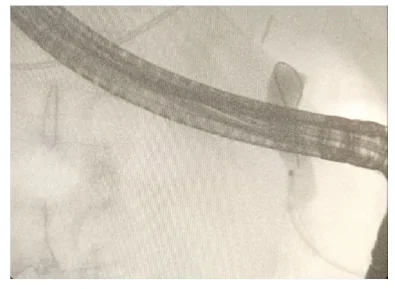
Cholangiogram showing the hydrophilic guidewire coiling at the biliary stricture.

The patient underwent percutaneous cholangiography that confirmed a complete biliary stricture with no contrast flow into the small bowel that could not be traversed with a 0.035-inch guidewire (Figure 3). The cholangioscopy was performed and the diathermic puncture was made with a 0.035-inch modified guidewire, removing the coating at both ends for current transmission. Difficulties were encountered when attempting to traverse the stricture with the 0.035-inch flexible guidewire tip. Downsizing to a 0.018-inch wire allowed passage. A needle knife was loaded over the guidewire, and electrocautery was applied at the stricture site; subsequently, a 0.035-inch guidewire was successfully passed.

**FIGURE 3:**
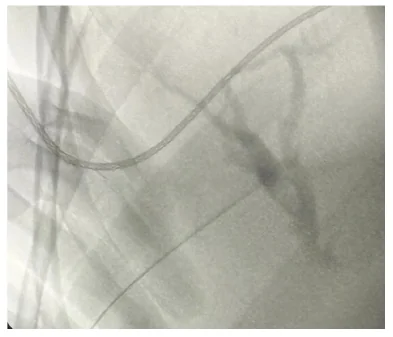
Percutaneous cholangiography showing a complete biliary stricture with no contrast flow into the small bowel.

However, the narrow lumen of the stricture prevented the passage of other instruments, including a 4 mm Soehendra dilator. Successful recanalization was achieved using the 7F and 10F Soehendra stent retrievers. A 10 mm x 6 cm fully covered self-expanding metallic biliary stent was then placed and secured with an endoscopic clip (Figure 4).

**FIGURE 4:**
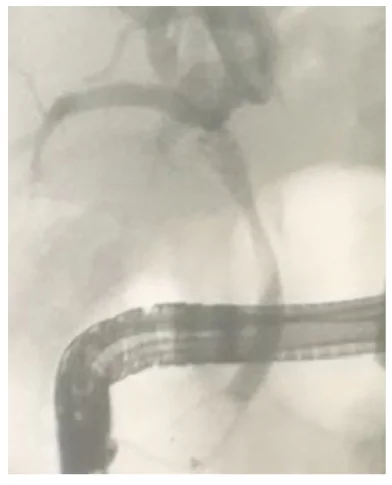
Correct passage of contrast through the recanalized stenosis after stent placement.

The patient recovered uneventfully, with no immediate or delayed complications following the procedure. Liver function tests normalized within two weeks. Outpatient follow-up remains ongoing, and stent removal is planned for twelve months post-procedure.

Endoscopic recanalization of complete biliary strictures has been previously reported using combined percutaneous and endoscopic approaches^1^. A standard needle knife has been employed for cutting and puncturing these strictures^2^. Ramzi Mulki et al. demonstrated cholangioscopic guidance to enable controlled cutting with a needle knife^3^.

This is the first report of endoscopic treatment for complete biliary stenosis using diathermic puncture with a modified guidewire, followed by recanalization with a Sohendra extractor. This minimally invasive technique could be an alternative to surgical repair for complete and short benign biliary strictures.

## Data Availability

Research data are presented within the article itself, including Figures 1, 2A, 2B, 2C, and the video material
